# Dataset of hygrothermal and energy measurements for a raw compressed earth brick house of Sense-City equipment during different seasons

**DOI:** 10.1016/j.dib.2025.111405

**Published:** 2025-02-19

**Authors:** Julien Waeytens, Myriam Duc, Yan Ulanowski, Laurent Ibos, Thibaut Colinart, Hadi Nasser, Mostafa Mortada, Hamza Allam, Abderrahim Boudenne, Nicolas Dujardin, Kamel Zibouche, Etienne Gourlay, Jean-Pierre Monchau, Fionn McGregor

**Affiliations:** a Université de Gustave Eiffel, Champs-sur-Marne, F-77420, France; b Université de Paris-Est Créteil, CERTES, Creteil, F-94000, France; c Université de Bretagne Sud, UMR CNRS 6027, IRDL, Lorient, F-56100, France; d Centre Scientifique et Technique du Batiment (CSTB), Champs-sur-Marne, F-77420, France; e Cerema, BPE Research Team, Strasbourg, F-67035, France; f Themacs Ingenierie, Champs-sur-Marne, F-77420, France; g Université de Pau et des Pays de l'Adour, E2S UPPA, SIAME, Anglet, F-64600, France

**Keywords:** Building demonstrator, Indoor comfort monitoring, Hygrothermal behaviour, Earth construction, Compressed Earth block

## Abstract

A non-insulated raw compressed earth brick test house was built and deeply instrumented in Sense-City equipment at Champs sur Marne, France. A wide variety of sensors were deployed in the room, inside the walls and outside to monitor the hygrothermal behaviour, the thermal comfort and the heating energy consumption. The measurement campaigns were performed over several weeks during winter, spring and summer seasons in 2024. The test house was unoccupied and exposed to natural weather conditions. During winter tests, different heating scenarios were considered. The provided dataset of sensor outputs can be useful for a better understanding of earthen construction and for the experimental validation of building physics models at both the wall and building scales.

Specifications Table

**Table:** 

Subject	Engineering.
Specific subject area	Monitoring of the hygrothermal behaviour, the thermal comfort and the heating energy consumption of a non-insulated raw compressed earth brick (CEB) test house under natural weather conditions.
Type of data	Table (.csv format), Plan, Revit file Raw, Processed.
Data collection	The list of sensor outputs collected, at a 1-minute acquisition time step, in the raw compressed earth brick house of Sense-City equipment at Université Gustave Eiffel is: - Surface temperature, CS241 model (Campbell Scientific) - Temperature & relative air humidity, DKRF400 model (Driesen + Kern) and Hygrovue10 model (Campbell Scientific) - Heat flux (CAPTEC) - Black globe temperature (Campbell Scientific) - Air velocity sensor, Windsonic model (Gill) - Net radiometer, NR01 model (Hukseflux) - Rain gauge, ARG314 model (Campbell Scientific).
Data source location	Institution: Université Gustave Eiffel, Sense-City Equipment City: Champs sur Marne Country: France Latitude and Longitude for collected samples: 48.841760, 2.589967.
Data accessibility	Repository name: Data_CEB_SenseCity_2024 Data identification number: https://doi.org/10.57745/PTGKSM Direct URL to data: https://entrepot.recherche.data.gouv.fr/dataset.xhtml?persistentId=doi:10.57745/PTGKSM The data are hosted on the French website “Recherche Data Gouv – Université Gustave Eiffel”. Data can be accessed via the DOI given above and are stored in a zip file.
Related research article	

## 1 Value of the Data

• Several building physics quantities, *e.g.* temperature, relative humidity and heating energy consumption, were measured in a real non-insulated raw compressed earth brick house under different weather conditions (winter, spring and summer).
• Sensor data were collected in the room, on the wall surface, inside the walls and outside.
• The dataset can be useful for researchers, engineers and students to test and to validate modelling and simulation at wall and at building scales.
• The dataset can be valuable to study and to promote the hygrothermal behavior and the thermal comfort of earthen construction.

## 2 Background

This work is carried out in the context of improving building energy efficiency and reducing the carbon footprint through the use of natural building materials. Earthen materials offer numerous advantages but also present certain challenges [[1], [2], [3]]. Many studies highlight the complexity of accounting for the hygrothermal behaviour of building materials, as well as the scarcity of experimental data at the building scale. This issue is particularly relevant for earthen materials, such as compressed earth bricks, due to their high hygroscopic properties which influence the indoor thermal comfort [[4], [5], [6], [7], [8], [9]].

**Fig. 1:**
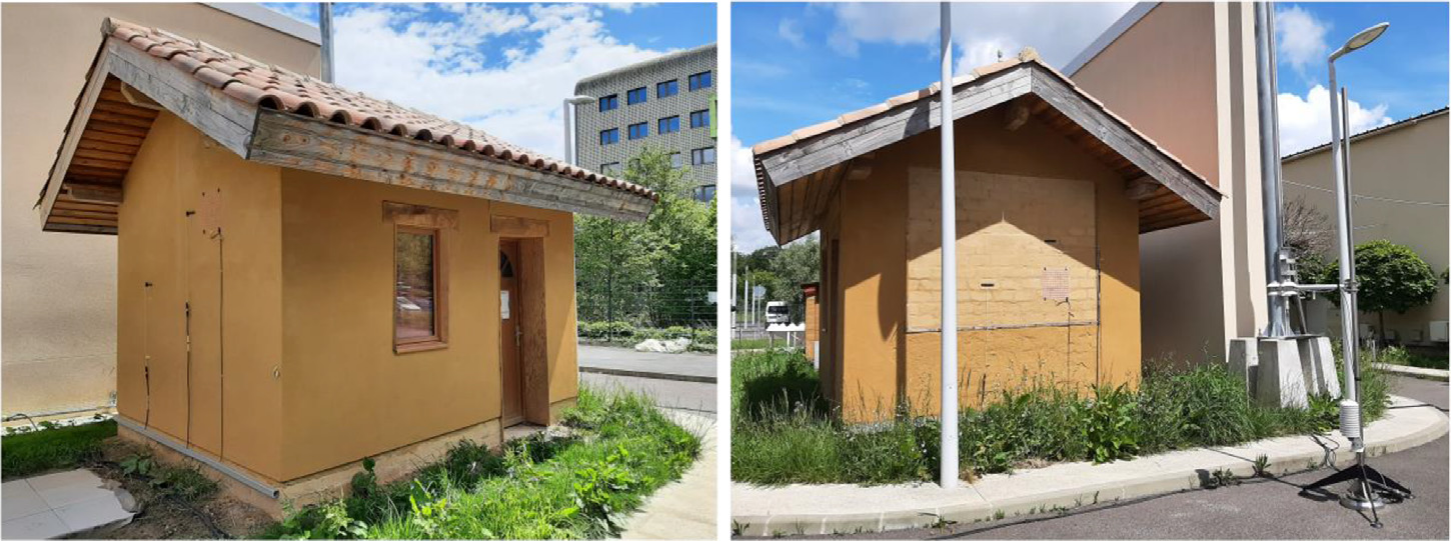
Pictures of the raw compressed earth brick house in May 2024: north-west view (at left) and south view (at right).

The studied raw compressed earth brick (CEB) test house of 12 m^2^ (see Fig. 1) was built in 2020 in the Sense-City equipment at Université Gustave Eiffel, Champs sur Marne (France) for research purposes. In fact, Sense-City equipment consists of two small districts of 400 m^2^ at full scale to study innovative and sustainable solutions for the city of tomorrow. Thanks to a huge climatic chamber mounting on rails, experimental tests can be conducted under controlled conditions (temperature, relative humidity, sun lamp, rain) or in natural weather conditions by removing the climatic chamber. The Sense-City districts are equipped with a large number and variety of sensors.

Originally, the measurements obtained in the non-insulated CEB house were intended for research in the French National Research project RESBIOBAT focusing on the in-situ thermal resistance identification of hygroscopic walls and in the French National Project on earth (PN Terre) addressing issues related to modelling, simulation, experiment and durability. Given the lack of data on earthen construction at both the wall and building scales, the goal of this article is to provide to the scientific community a well-documented dataset of sensor outputs on the CEB house for studying notably the hygrothermal behaviour, the thermal comfort and energy consumption purposes.

## 3 Data Description

The main data folder is named “Dataset_CEBSenseCity_2024”. In the main data folder, the detailed map of the house in pdf format (“Map_CEBhouse.pdf”) and its geometric model in REVIT format (“CEB_revit.rvt”) are provided. This folder has 5 subfolders using the nomenclature “Data_CEB_Ti_Date” where i={1,..,5} is the experiment test number and Date indicates the month and the year of the test.

• “Data_CEB_T1_022024” and “Data_CEB_T2_022024” are associated with the 2 winter tests
• “Data_CEB_T3_042024” and “Data_CEB_T4_042024” are associated with the 2 spring tests
• “Data_CEB_T5_07082024” corresponds to the summer test from July to August 2024.

In each subfolder, the sensor outputs are given in Tables in .csv format:

- “rawdata_Ti_Heating.csv” gives the raw data of heating from the connected outlet for the test Ti, i={1,..4}
- “data_Ti_dt1min.csv” summarized all the recorded sensor outputs at a 1 min time step during the test Ti, i={1,..5}.

**Fig. 2:**
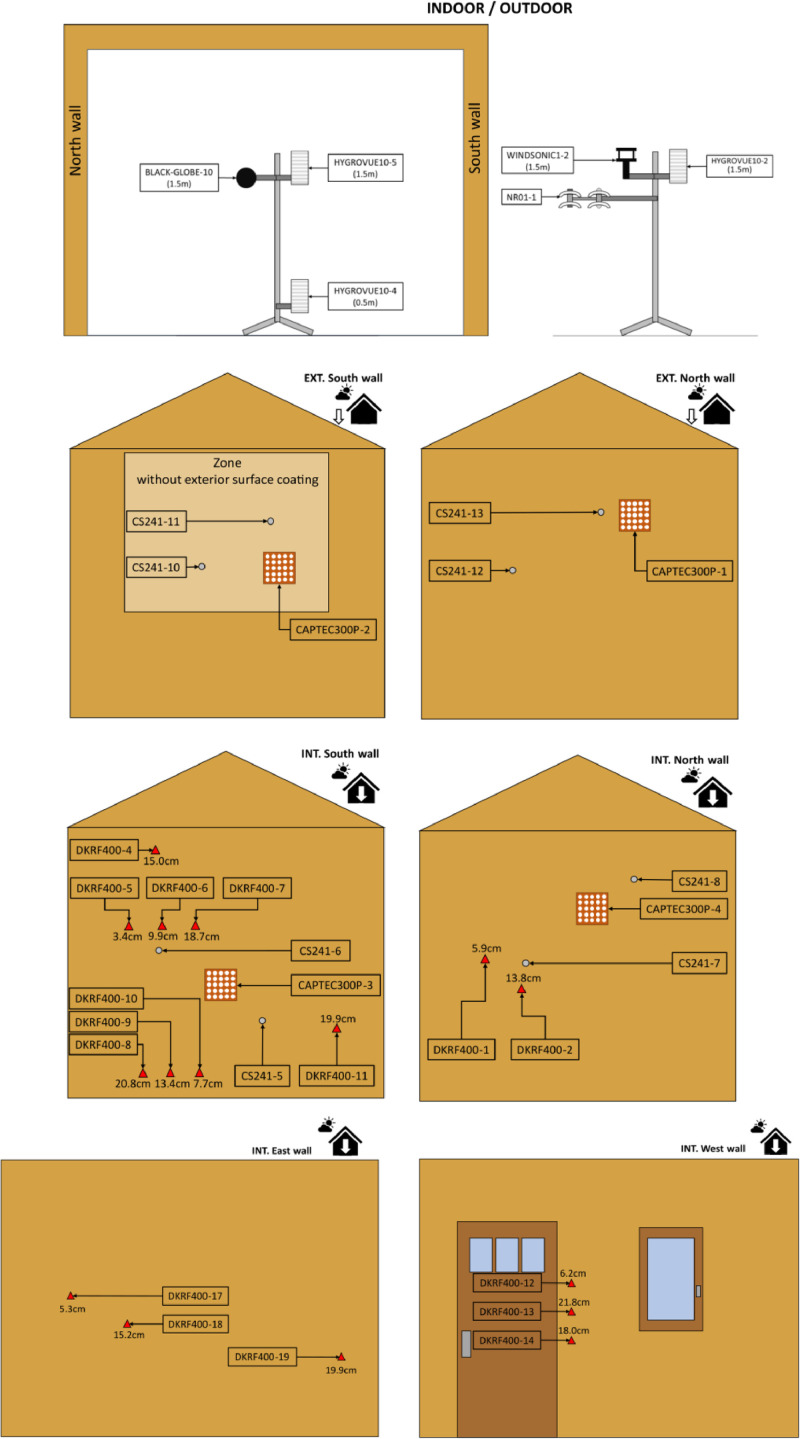
Instrumentation plan and sensor names of the CEB house – the value in cm next to the temperature/humidity sensors indicates the depth of the sensor in the wall from the inside surface.

The date and time are in the first column and the measured physical quantities are in the subsequent columns. The sensors, whose instrumentation plan is represented in Fig. 2, are designated in .csv Tables as follows:

In the room of CEB house

- HygroVUE_10_x_Temperature (°C): Air temperature in the room measured by HygroVUE sensor, at 0.5 m height for x=4 and at 1.5m height for x=5
- HygroVUE_10_x_Humidity (%): Air Relative Humidity in the room measured by HygroVUE sensor, at 0.5 m height for x=4 and at 1.5m height for x=5
- HygroVUE_10_x_Absolute_Humidity (g/m^3^): Absolute humidity in the room calculated from HygroVUE sensor, at 0.5 m height for x=4 and at 1.5m height for x=5
- HygroVUE_10_x_Vapor_Pressure (Pa): Water vapor pressure in the room calculated from HygroVUE sensor, at 0.5 m height for x=4 and at 1.5m height for x=5
- Black_Globe_10 (°C): Black globe temperature in the house room at 1.5 m height
- SO_A240_Power (W): Power consumption of electrical heating in the room
- SO_A240_Energy_Today (kW.h): Cumulative energy consumption on each day of electrical heating in the room

On wall surfaces and inside walls

- CS241_x (°C): Surface temperature on inside walls, x={5,..,8}
- CS241_x (°C): Surface temperature on outside walls, x={10,..,13}
- CAPTEC300P_x_Flux (W/m2): Heat flux of 300 mm by 300 mm on inside and outside wall surfaces, x={1,..,4} – in the chosen convention, a heat flux directed from outside to inside (resp. from inside to outside) is noted positively (resp. negatively).
- DKRF400_x_Temperature (°C): Temperature measured by DKRF400 sensor placed inside the walls, x={1,2,4,..,14,17,18,19}
- DKRF400_x_Humidity (%): Air Relative Humidity measured by DKRF400 sensor placed inside the walls, x={1,2,4,..,14,17,18,19}
- DKRF400_x_Absolute_Humidity (g/m^3^): Absolute humidity calculated from DKRF400 sensor placed inside the walls, x={1,2,4,..,14,17,18,19}
- DKRF400_x_Vapor_Pressure (Pa): Water vapor pressure calculated from DKRF400 sensor placed inside the walls, x={1,2,4,..,14,17,18,19}

The height and depth position of the sensors in walls are given in Table 1.

**Table 1:** Height and depth position of sensors in CEB walls from the inside surface.

Denomination	Height and depth in the wall	Denomination	Height and depth in the wall
CS241_5	1.00 m-0 cm	CS241_6	1.60 m-0 cm
CS241_7	1.45 m-0 cm	CS241_8	2.15 m-0 cm
CS241_10	1.40 m-30 cm	CS241_11	1.90 m-30 cm
CS241_12	1.55 m-30 cm	CS241_13	2.10 m-30 cm
CAPTEC300P_1_Flux	2.10 m-30 cm	CAPTEC300P_2_Flux	1.45 m-30 cm
CAPTEC300P_3_Flux	1.30 m-0 cm	CAPTEC300P_4_Flux	1.90 m-0 cm
DKRF400_1	1.50 m-5.9 cm	DKRF400_2	1.25 m-13.8 cm
DKRF400_4	2.40 m – 15 cm	DKRF400_5	1.80 m-3.4 cm
DKRF400_6	1.80 m-9.9 cm	DKRF400_7	1.80 m-18.7 cm
DKRF400_8	0.58 m-20.8 cm	DKRF400_9	0.58 m-13.4 cm
DKRF400_10	0.58 m-7.7 cm	DKRF400_11	1.00 m-19.9 cm
DKRF400_12	1.50 m-6.2 cm	DKRF400_13	1.25 m-21.8 cm
DKRF400_14	1.00 m-18 cm	DKRF400_17	1.50 m-5.3 cm
DKRF400_18	1.25 m-15.2 cm	DKRF400_19	1.00 m-19.9 cm

Outdoor weather

- HygroVUE_10_2_Temperature (°C): outside air temperature measured at 1.5 m height close to the south-oriented wall
- HygroVUE_10_2_Humidity (%): outside air relative humidity measured at 1.5 m height close to the south-oriented wall
- HygroVUE_10_2_Absolute_Humidity (g/m^3^): outside absolute humidity calculated from HygroVUE sensor, at 1.5 m height close to the south-oriented wall
- HygroVUE_10_2_Vapor_Pressure (Pa): outside water vapor pressure calculated from HygroVUE sensor, at 1.5 m height close to the south-oriented wall
- WindSonic1_2_Wind_Direction (°): outside wind direction measured at 1.5 m height close to the south-oriented wall
- WindSonic1_2_Wind_Speed (m/s): outside wind velocity measured at 1.5 m height close to the south-oriented wall
- NR01_1_SR01_Up (W/m2): Short wave solar irradiance (direct+diffuse) measured horizontally at 1.5 m height close to the south-oriented wall
- HygroVUE_10_3_Temperature: outside air temperature measured at 7 m height in Sense-City district
- HygroVUE_10_3_Humidity: outside air relative humidity measured at 7 m height in Sense-City district
- HygroVUE_10_3_Absolute_Humidity (g/m^3^): outside absolute humidity calculated from HygroVUE sensor, at 7 m height in Sense-City district
- HygroVUE_10_3_Vapor_Pressure (Pa): outside water vapor pressure calculated from HygroVUE sensor, at 7 m height in Sense-City district
- WindSonic1_3_Wind_Direction (°): outside wind direction measured at 7 m height in Sense-City district
- WindSonic1_3_Wind_Speed (m/s): outside wind velocity measured at 7 m height in Sense-City district
- ARG314_Rain (mm): Rain precipitation measured in Sense-City district
- ClimaVUE_50_1_Air_temperature (°C): outside air temperature measured at 7 m height by Sense-City weather station
- ClimaVUE_50_1_Air_pressure (Pa): outside air pressure measured at 7 m height by Sense-City weather station
- ClimaVUE_50_1_Humidity (%): outside air relative humidity measured at 7 m height by Sense-City weather station
- ClimaVUE_50_1_Vapor_Pressure (hPa): Water vapor pressure measured at 7 m height by Sense-City weather station
- ClimaVUE_50_1_Solar_flux (W/m2): Solar irradiance (direct+diffuse) measured at 7 m height by Sense-City weather station
- ClimaVUE_50_1_Wind_Direction (m/s): outside wind direction measured at 7 m height by Sense-City weather station
- ClimaVUE_50_1_Wind_Speed (m/s): outside wind velocity measured at 7 m height by Sense-City weather station
- ClimaVUE_50_1_Precipitation (mm): Rain precipitation measured at 7 m height by Sense-City weather station.

Let us note that the data provided in this article for HYGROVUE and DKRF400 sensors have been corrected using a linear formula whose gain and offset are given in Tables 2 and 3.

**Table 2:** Corrective coefficient for temperature sensor outputs.

Denomination	Gain a	Offset b (°C)
DKRF400-1	0.9915	0.1094
DKRF400-2	1.0101	-0.3624
DKRF400-3	1.0087	-0.4261
DKRF400-4	1.0205	-0.6453
DKRF400-5	1.022	-0.88
DKRF400-6	0.9858	0.0971
DKRF400-8	1	-0.16
DKRF400-9	0.9886	0.0898
DKRF400-10	0.9929	-0.383
DKRF400-11	1.0087	-0.4873
DKRF400-12	1.0058	-0.3273
DKRF400-13	0.9928	-0.1547
DKRF400-14	1.0029	-0.5894
DKRF400-17	1.0058	-0.408
DKRF400-18	1	-0.52
DKRF400-19	0.9943	-0.2036
DKRF400-20	1.0282	-1.1285
HygroVUE10-4	1	0
HygroVUE10-5	1	0

**Table 3:** Corrective coefficient for relative humidity sensor outputs.

Denomination	Gain a	Offset b (%)
DKRF400-1	1.137	-4.03
DKRF400-2	1.124	-4.11
DKRF400-3	1.128	-3.13
DKRF400-4	1.155	-4.16
DKRF400-5	1.12	-3.31
DKRF400-6	1.095	-2.27
DKRF400-8	1.23	-13.04
DKRF400-9	1.24	-12.65
DKRF400-10	1.229	-11.37
DKRF400-11	1.145	-4.24
DKRF400-12	1.125	-4.3
DKRF400-13	1.117	-3.85
DKRF400-14	1.127	-3.51
DKRF400-17	1.136	-4.04
DKRF400-18	1.114	-3.36
DKRF400-19	1.122	-3.46
DKRF400-20	1.156	-4.66
HygroVUE10-4	1.08	-2.69
HygroVUE10-5	1.069	-2.13

## 4 Experimental Design, Materials and Methods

The non-insulated raw compressed earth brick (CEB) house, measuring 12 m² (3 m × 4 m), was built in 2020. The house rests on a 15 cm-thick concrete slab. The walls are constituted of 30cm-thick compressed earth bricks and an exterior raw earth plaster of about 1.5 cm (no rendering inside). As shown in Fig. 1, a part of the south-oriented wall was left without exterior raw earth plaster for future research purposes (see detailed map in pdf format). Concerning the roof with 22 m^2^ apparent surface, wood was used as traditional building material. Two wood fiber insulating panels, measuring 12 cm and 4 cm in thickness respectively, were installed on 2 cm thick wooden sarking boards. Fired large canal tiles, commonly used in southern France, were installed on the roof, sealed with hydraulic lime, without edge tiles. Wood plates formed an aesthetic formwork all around the construction and the roof overhang is 60 cm (for rain protection) on the 4 sides. The house has one window and one door, both located on the west-oriented wall. There is no ventilation system in the house. The detailed map of the test house is provided in the data article in pdf format. The west-oriented wall (resp. east-oriented wall) is at a distance of 1.40 m from a tall cavity wall of 6 m height constituted of building blocks (resp. a distance of 12 m from the wall of the Sense City climatic chamber, which is 11 m high. Raw compressed earth bricks material characteristics and more details on CEB house construction can be found in [10].

In all the experimental tests (T1, T2, T3, T4 and T5), the house was unoccupied, no additional moisture was introduced in the room. Natural weather conditions were considered (no use of Sense-City climatic chamber). The door and a window curtain were kept close during all the time period of the tests. During the winter (T1, T2) and spring tests (T3, T4) an oil-filled heater was placed at the center of the room. The heating electrical energy consumption was recorded using a connected outlet. In these tests (T1, T2, T3, T4), a low-speed fan (air velocity of 0.15m/s measured by a 1D hot wire anemometer at the center of the room) was used to homogenize the air temperature in the room. The specificities of each test are detailed below:

• T1 – Winter test from January 25 to February 8 2024 – A 20°C setpoint on the whole time period has been programmed for heating. Let us note that before starting the first test T1, the CEB house had not been heated.
• T2 – Winter test from February 9 to February 25 2024 – For each of these days, a 20°C setpoint was set between 6am and 10pm and heating was turned off (free cooling) during the night from 10pm to 6am.
• T3 – Spring test from April 3 to April 17 2024 – A 20°C setpoint on the whole time period has been programmed for heating. Let us note that the CEB house was not heated during the month of March 2024.
• T4 – Spring test from April 17 to May 2 2024 – For each of these days, a 20°C setpoint was set between 6am and 10pm and heating was disactivated (free cooling) during the night from 10pm to 6am.
• T5 – Summer test from July 12 to September 1 2024 – During this summer period, no fan was used in the room. The door and window were kept close.

The CEB house was instrumented in 2023 with a wide variety of sensors (see DATA COLLECTION and DATA DESCRIPTION sections). The instrumentation map is represented in Fig. 2. For more information on the technical processes to place temperature/relative humidity in the earth walls and the heat flux sensors, the reader can refer to [10]. All the sensors were connected by wire to a CR1000X acquisition card or its VOLT 116 module extension. The sensor outputs collected on the acquisition card were sent by WIFI to STOCO Sense-City server. The provided data in this article are an extraction from STOCO Sense-City server during the test periods from winter to summer 2024.

Before deployment, the temperature and air relative humidity sensors were checked and calibrated in May 2023 at the metrology department of Université Gustave Eiffel. A linear correction formula was provided for each temperature and relative humidity sensor and was implemented in the acquisition card's code:

$$y_{c}=a\times y_{r}+b$$

where $y_{r}$, $y_{c}$ are the read and the corrected values respectively, and a and b are the corrective coefficient (gain and offset) determined by the metrology department. The corrective coefficients for temperature sensors are given in Table 2, and for relative humidity sensors in Table 3. In the present article, we provide the corrected measurement values for the sensors DKRF400 and HygroVUE104-5.

Then, from corrected temperature T and relative humidity RH sensor outputs, additional physical quantities have been calculated such as absolute humidity A (g/m3) and water vapour pressure p_v_ (Pa), using the following formula from ISO 13788 [11]:

$$p_{v}=\frac{RH}{100}p_{vs}\;where\;\left\{ \begin{array}{c} p_{vs}=610.5\;exp\left(\frac{17.269\;T}{237.3+T}\right),\;if\;T\geq 0^{\circ }C\\ p_{vs}=610.5\;exp\left(\frac{21.875\;T}{265.5+T}\right),\;if\;T<0^{\circ }C \end{array}\right.$$

$$A=\;\frac{M_{H20}\times p_{v}}{R\times \;\left(273.15+T\right)}=\frac{18.0154\;\times p_{v}}{8.3145\;\times \;\left(273.15+T\right)}$$

## Limitations

The dataset was obtained from sensors that are affected by measurement error. For more details on the accuracy of the sensors used, please refer to [10].

During the winter and spring experimental tests (T1, T2, T3, T4), the outdoor weather conditions at 7 m height in Sense-City were monitored using HygroVUE10_3 and Windsonic_1_2 sensors. Then, in the summer test T5, these outdoor sensor at 7 m height were replaced by the weather station ClimatVUE_50_1.

The door and window were kept closed throughout all the tests, except on August 22 2024 in summer test T5.

Lastly, due to WIFI communication issues, some sensor outputs were lost. It only concerns the electrical heating consumption from February 11 at 11:55am to February 15 2024 at 9:54am during winter test T2 and WindSonic_1_3 outputs at the end of the spring test T4 from May 1 at 7:53pm to May 2 2024 at 10pm.

## Ethics Statement

The authors have read and follow the ethical requirements for publication in Data in Brief and confirming that the current work does not involve human subjects, animal experiments, or any data collected from social media platforms.

## CRediT Author Statement

**Julien Waeytens**: Conceptualization, Methodology, Formal analysis, Investigation, Resources, Visualization, Supervision, Project administration, Funding acquisition, Writing – Original Draft, **Myriam Duc**: Conceptualization, Resources, Supervision, Project administration, Funding acquisition, Writing – Original Draft, **Yan Ulanowski**: Methodology, Investigation, Resources, **Laurent Ibos**: Conceptualization, Methodology, Formal analysis, Writing – Original Draft, **Thibaut Colinart**: Conceptualization, Methodology, Formal analysis, Writing – Original Draft, **Hadi Nasser**: Investigation, **Mostafa Mortada**: Investigation, **Hamza Allam**: Conceptualization, Methodology, Writing – Review & Editing, **Abderrahim Boudenne**: Conceptualization, Methodology, Writing – Original Draft, **Nicolas Dujardin**: Investigation, Writing – Review & Editing, **Kamel Zibouche**: Conceptualization, Methodology, Writing – Review & Editing, **Etienne Gourlay**: Conceptualization, **Jean-Pierre Monchau**: Methodology, **Fionn McGregor**: Conceptualization, Methodology, Resources, Project administration, Funding acquisition, Writing – Original Draft.

## Data Availability

DataverseDataset of hygrothermal and energy measurements for a raw compressed earth brick house of Sense-City equipment during different seasons (Reference data)
